# Foreign body associated non-ST elevation myocardial infarction: a case report

**DOI:** 10.15171/jcvtr.2017.39

**Published:** 2017-12-20

**Authors:** Pooyan Dehghani, Mahdi Sajedi Khanian

**Affiliations:** ^1^Department of Cardiology, School of Medicine, Shiraz University of Medical Sciences, Shiraz, Iran; ^2^Cardiovascular Research Center, Shiraz University of Medical Sciences, Shiraz, Iran; ^3^Department of Cardiology, School of Medicine, Sabzevar University of Medical Sciences, Sabzevar, Iran

**Keywords:** Foreign Body, Broken Guiding Catheter Tip, Non-ST Elevation Myocardial Infarction

## Abstract

The incidence of guiding catheter tip fracture, is quite rare during percutaneous coronary interventions (PCI). Retained broken catheter tip in coronary artery and circulation can result in serious complications such as thrombosis, embolism, acute occlusion and myocardial infarction, arrhythmias and sepsis. It is mandatory to remove the fractured catheter as soon as possible to avoid these complications. Transcatheter removal is the treatment of choice. If percutaneous removal is failed, surgical intervention is advised.

## Introduction

The incidence of broken equipments, especially the fracture of guiding catheter tip, is quite rare during percutaneous coronary interventions (PCI).^1,2^ In most cases the fracture is incomplete and a partially broken catheter can be removed as a whole. When complete dehiscence and embolization happens, it can be either snared from the coronary tree^3^ or removed by using a low profile balloon inflated distal to the embolized tip.^4^ When these maneuvers are not successful, surgical intervention is advised. It should be however mentioned that the most important issue here is diagnosing the problem at first hand. In this paper, we present a case with undiagnosed complete fracture of guiding catheter tip during PCI which led to subacute coronary thrombosis after 37 days.

## Case Presentation

**Figure 1:**
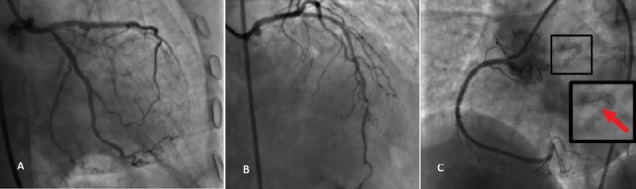
Successful percutaneous coronary intervention on both left anterior descending (A,B) and right coronary arteries (C). *Note the fractured tip of the guiding catheter in the left main coronary artery during right coronary artery injection [Arrow].

**Figure 2:**
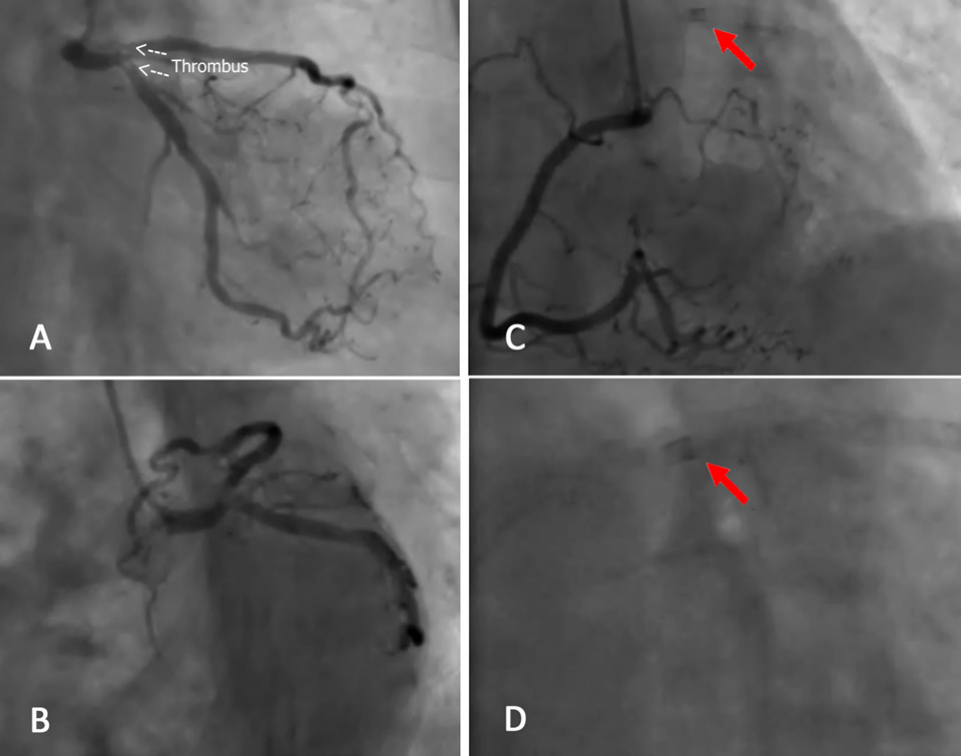
Coronary thrombosis in left main coronary artery with extension to both left anterior descending and left circumflex arteries (A,B). The fractured tip of the guiding catheter in left main coronary artery during right coronary artery injection [Arrow] (C) The fractured tip of the guiding catheter in AP view [Arrow] (D)

A 65-year-old female was initially admitted with typical angina and impression of acute coronary syndrome in another center. She only had a medical history of hypertension. The electrocardiogram (ECG) had shown 2 mm T inversion in V1-V3 leads, biphasic t waves in I, aVL and QT prolongation (QTc: 0.520 seconds). In the hospital coronary angiography was performed which had revealed two vessel disease. Successful percutaneous coronary intervention was done on left anterior descending artery (LAD) and right coronary artery (RCA) in the same session (Figure 1). The patient was discharged home the next day. She was doing well for about a month till 2 days prior to her second admission when presented with multiple episodes of angina on exertion. She was admitted again. Cardiac enzymes were within normal limits and there were no new ECG changes. Echocardiography showed left ventricular ejection fraction of 55% without any resting wall motion abnormalities. She still complained of occasional angina pain until she developed a severe resting chest pain associated with nausea, vomiting and diaphoresis at the second day of admission. There was dynamic ECG changed associated with a rise in cardiac biomarkers. The patient was transferred to our center for urgent coronary angiography. In our emergency department, her angina had already decreased to 3 out of a 10 scale pain score. Electrocardiography showed ST depression in V2-V5 leads. The patient was sent to catheterization unit and the problem was observed just before contrast injection. There was a foreign body material stuck in the left main coronary artery which was the tip of a guiding catheter. Coronary angiography showed patent RCA and LAD stents, but there was a significant thrombotic narrowing of distal left main artery with extension of thrombus to the proximal parts of both LAD and left circumflex arteries because of the tip of a guiding catheter lodged in the left main coronary artery (Figure 2 and Video 1). The patient was sent immediately to the operating room and coronary artery bypass grafting was done successfully. Only two saphenous vein grafts on LAD and main obtuse marginal branches were anastomosed without any attempt to remove the foreign body. The patient was discharged from hospital after 3 days without any further complications.

## Discussion

Intravascular fracture and dehiscence of angiography catheters is quite rare and there are only a few reports in the literature in this regard.^1,4^ One of the most common sites of catheter fractures is at the site of connecting the shaft with the tip which leads to dehiscence and even embolization of the catheter tip.^2,4^ Excessive torquing and poor handling of catheters, manufacturing errors or combination of these could lead to fracture of the catheters. Other causes of fractures are reuse of catheters, polymer aging, and forceful withdrawal of catheter which is then stuck in an arterial spasm.^5^

Retained broken catheter tip in coronary artery and circulation can result in serious complications such as thrombosis, embolism, acute occlusion and myocardial infarction, arrhythmias and sepsis.^3^ So it is mandatory to remove the fractured catheter as soon as possible to avoid these complications. Transcatheter removal is the treatment of choice. Devices used for retrieval includes nitinol goose neck snares, baskets, bioptomes and balloon catheters. If percutaneous removal fails, surgical intervention is mandatory.

There are several reports of successful transcatheter removal of fractured angiographic catheters in the literature. These include fracture of tip of diagnostic and guiding catheters and even shaft of the guiding catheters.^2,3,6,7^ In all of the presented cases the problem was diagnosed at the time of intervention.

In our case, unfortunately the embolization of the fractured catheter tip was not diagnosed at the time of intervention which led to coronary thrombosis after 37 days. When we carefully reviewed the angioplasty procedure, we recognized the presence of the fractured tip in the left main coronary artery while RCA angioplasty was being performed (Figure 1-C). If the problem was diagnosed at that time because of its coaxial position, a guidewire could be easily passed through the fractured tip, then after passing an appropriate sized balloon through the lumen of the broken catheter tip, the balloon could be inflated distal to the catheter tip and an attempt could be made to pull back the balloon and the fractured tip as a whole into the guiding catheter. Nevertheless in his second admission due to the presence of thrombus which extended to proximal parts of LAD and LCX, we did not take the risk to make an attempt to remove the fractured tip and preferred to send the patient directly for coronary artery bypass grafting surgery.

So we suggest that interventional cardiologists carefully check the entire coronary tree fluoroscopically and also the tip of guiding catheter at the end of their procedures to be certain that no foreign bodies including any remnant of guiding catheters, guide wires, etc. remain in the patient’s circulation.

## Ethical approval

The patient had signed the informed consent form.

## Competing interests

All authors declare no competing financial interests exist.

## Supplementary Materials

Supplementary Materials Supplementary file contains a video file.
